# Mechanism of Nano-Structuring Manipulation of the Crystallization Temperature of Superlattice-like [Ge_8_Sb_92_/Ge]_3_ Phase-Change Films

**DOI:** 10.3390/nano11010020

**Published:** 2020-12-24

**Authors:** Qingqian Qiu, Pengzhi Wu, Yifeng Hu, Jiwei Zhai, Tianshu Lai

**Affiliations:** 1 State-Key Laboratory of Optoelectronic Materials and Technologies, School of Physics, Sun Yat-Sen University, Guangzhou 510275, China; chouqq@mail2.sysu.edu.cn (Q.Q.); winlanwu@126.com (P.W.); 2 Functional Material Research Laboratory, Tongji University, Shanghai 200092, China; hyf@jsut.edu.cn

**Keywords:** phase-change memory, superlattice-like (SLL) structure, coherent phonon spectroscopy, stress, Raman spectroscopy

## Abstract

Superlattice-like (SLL) phase-change film is considered to be a promising phase-change material because it provides more controllabilities for the optimization of multiple performances of phase-change films. However, the mechanism by which SLL structure affects the properties of phase-change films is not well-understood. Here, four SLL phase-change films [Ge_8_Sb_92_(15 nm)/Ge (x nm)]_3_ with different x are fabricated. Their behaviors of crystallization are investigated by measuring sheet resistance and coherent phonon spectroscopy, which show that the crystallization temperature (*T*_C_) of these films increases anomalously with x, rather than decreases as the interfacial effects model predicted. A new stress effect is proposed to explain the anomalous increase in *T*_C_ with x. Raman spectroscopy reveals that Raman shifts of all phonon modes in SLL films deviate from their respective standard Raman shifts in stress-free crystalline films, confirming the presence of stress in SLL films. It is also shown that tensile and compressive stresses exist in Ge and Ge_8_Sb_92_ layers, respectively, which agrees with the lattice mismatch between the Ge and Ge_8_Sb_92_ constituent layers. It is also found that the stress reduces with increasing x. Such a thickness dependence of stress can be used to explain the increase in crystallization temperature of four SLL films with x according to stress-enhanced crystallization. Our results reveal a new mechanism to affect the crystallization behaviors of SLL phase-change films besides interfacial effect. Stress and interfacial effects actually coexist and compete in SLL films, which can be used to explain the reported anomalous change in crystallization temperature with the film thickness and cycle number of periods in SLL phase-change films.

## 1. Introduction

In recent years, phase-change memory (PCM) has been recognized as one of the promising next generations of memory devices because PCM may have many advantages over current static random access memory (SRAM), dynamic random access memory (DRAM), and flash memory devices as well as optical DVD-ReWritable disc memory, including high speed, good thermal stability, non-volatility, low power consumption, good scalability from micro- to nano-meter cell size, and good compatibility with Si-based Complementary Metal Oxide Semiconductor (CMOS) techniques [1,2,3,4]. Beyond-von-Neumann computing has been proposed as well based on phase-change memory devices [5]. However, such numerous advantages are usually difficult to be fulfilled simultaneously by a simple phase-change material. For example, the GeSbTe phase-change material used currently has some drawbacks, such as a slow crystallization rate, poor thermal stability, a higher writing power, etc. Although Sb-rich Ge_1−x_Sb_x_ (x > 0.9) phase-change films were found to have a high optical reflectivity contrast between amorphous and crystalline states and a growth-dominant crystallization mechanism that led to a fast crystallization rate, they had poor thermal stability or a short-time retention of data [6,7]. Therefore, different techniques have been developed to improve or optimize the properties of phase-change materials in past two decades. Doping phase-change materials with single elements or compounds has been used extensively to enhance both the thermal stability [8,9,10,11,12] and resistance of the crystalline state [11,12], and to reduce the crystallization time of phase-change materials. However, it was very difficult for doping alone to fulfill all advantages of phase-change memory or to optimize all properties of phase-change materials [8,9,10,11,12,13]. For example, nitrogen doping into GeSb could cause the change of the crystallization mechanism of doped GeSb films from growth-dominant to nucleation-dominant crystallization (slowing crystallization rate down), although it could enhance the thermal stability or raise crystallization temperature and increase the resistance of crystalline doped GeSb films [10]. Apparently, it is very necessary to develop new techniques that can provide more degrees of freedom simultaneously to optimize multiple performances of phase-change materials. Fortunately, superlattice-like (SLL) phase-change materials were proposed and realized in recent years [14]. Two different material films, where one or both films were phase-change material, were arranged alternately into a periodic multilayer structure—so-called superlattice-like (SLL) nanostructure films. Such an SLL nanostructure can provide more degrees of freedom to manipulate the properties of phase-change film materials, such as the periodic size and cycle number of SLL structures, the combination of two different component layers, and the thickness ratio of two different component layers composing each period of SLL structure. It has been reported that SLL structures could very effectively tune the crystallization temperature, thermal stability, the resistance in crystalline state, and thermal conductivity of the multilayer phase-change materials, and even lower the power assumption by changing the periodic size and thickness ratio of two constituent layers [15,16,17,18] as well as the cycle number of periods [19]. Therefore, SLL phase-change films were considered promising phase-change materials toward a low power consumption, high density data storage [20], and a good platform to optimize the multiple performances of phase-change materials due to multiple controllable degrees of freedom of SLL structures. Chong et al. [14] prepared the first SLL nano-films, [GeTe/Sb_2_Te_3_]_n_, where n was the number of the periodic unit. In that SLL film, GeTe/Sb_2_Te_3_ was 50 nm thickness and n varied between 4 and 12. The mixture of GeTe and Sb_2_Te_3_ in each basic unit corresponded to Ge_2_Sb_2_Te_5_. Authors fabricated two memory cells using [GeTe/Sb_2_Te_3_]_n_ and Ge_2_Sb_2_Te_5_ phase-change materials for comparison, respectively, and tested the SET (crystallization) and RESET (amorphization) processes using electrical pulses. It was found that SET and RESET switching currents as well as switching time became much smaller in the [GeTe/Sb_2_Te_3_]_n_ cell than in the Ge_2_Sb_2_Te_5_ cell. Authors attributed these merits of SLL [GeTe/Sb_2_Te_3_]_n_ films to the reduction in thermal conductivity in SLL structure with respect to Ge_2_Sb_2_Te_5_ films [14]. Thereafter, many SLL phase-change nano-films were fabricated and tested based on the combination of two different materials, such as [GeTe/Sb_7_Te_3_]_4_, [Si/Sb_80_Te_20_]_n_, [SiO_2_/Sb_80_Te_20_]_n_, and [Ge_8_Sb_92_/Ge]_n_, etc. [15,16,17,18]. It was found that the crystallization temperature of these SLL phase-change films could be tuned well in a wide range by changing the thickness ratio of two constituent layers and the period size of SLL films [15,16,17,18]. The tunability of crystallization temperature was explained by the interfacial effect that predicted the lowering of crystallization temperature with increasing film thickness [21,22]. However, some experimental phenomena could be explained neither by the interfacial effect nor by the reduction in thermal conductivity, such as the change in crystallization temperature with the cycle number of periodicity in SLL phase-change films [19] and the increase in crystallization temperature with the thickness of constituent layers in sandwich-structured phase-change films [8]. These exceptional phenomena implied that some new effects have not been exposed. Actually, the reduction in thermal conductivity was not essential to the explanation of the phenomenon of programming current decrease in SET and RESET processes of SLL films [14] because the lowering of crystallization temperature was observed in SLL phase-change films [23,24,25]. Furthermore, it was also observed that the resistance of SLL phase-change films increased in both amorphous and crystalline states [15,16,17,18], which certainly led to the decrease in a programming current in SET and RESET processes regardless of thermal conductivity. Therefore, the mechanism of SLL nano-structuring manipulation of the properties of SLL phase-change films is still unknown, or even controversial so far because a viewpoint of covalent- to resonant-state transition without an amorphization process, instead of crystalline to amorphous state transition, was also proposed [23].

In this paper, we design four SLL phase-change nanofilms, [Ge_8_Sb_92_ (15 nm)/Ge (x nm)]_3_, with variable thickness x of Ge layer (x = 2, 5, 8 and 11), and study the change in their crystallization behaviors using static heating together with the measurement of sheet resistance and femtosecond laser irradiation followed by in situ characterization of coherent phonon spectroscopy. Based on the two sets of measurement data, we believe that the crystallization temperature of the SLL films, [Ge_8_Sb_92_/Ge (x)]_3_, increases anomalously with x. Such an increase in crystallization temperature with x cannot be explained by the interfacial effect and/or the reduction in thermal conductivity. We propose a new mechanism: a stress effect to explain it. Lattice mismatch between two different constituent layers in SLL films and between sample films and substrates is common. As a result, stress should be ubiquitous in SLL films. Actually, Zacharias et al. already observed the presence of stress in ultra-thin films when they built up the model of interfacial effect [22]. However, the effect of stress on crystallization was not considered in their model. It was well known that stress could influence the crystallization of amorphous film materials. It was also reported that stress could enhance nucleation or reduce crystallization temperature [26,27,28]. Therefore, it is reasonable to take account for the effect of stress on crystallization as a new mechanism. Raman scattering spectroscopy is used to characterize the SLL samples [Ge_8_Sb_92_/Ge (x)]_3_ and monitor the shift of Raman scattering peaks of various phonon modes with x. We indeed observe the deviation of Raman scattering peak positions of various phonon modes from their standard Raman shift positions in stress-free bulk materials or thick films, which confirms the existence of stress in the SLL nano-film samples. Raman scattering peaks approach their standard Raman shift position in stress-free bulk materials with increasing x, revealing the reduction in or release of stress with increasing x. Such a reduction in stress with increasing x must lead to the rising of crystallizing temperature according to the phenomenon of stress-enhanced crystallization [18,19,20,21,22,23,24,25,26], which agrees well with our experimental observations. In other words, our experimental results can be explained well by a stress effect and reveal the important impact of stresses on the crystallization behaviors of SLL phase-change nanostructure films.

## 2. Materials and Methods

### 2.1. SLL Film Deposition

Superlattice-like films, [Ge_8_Sb_92_/Ge (x)]_3_ with three cycle periods and a fixed Ge_8_Sb_92_ layer thickness of 15 nm but variable thickness x of Ge layer, are deposited on glass substrates at room temperature by radio-frequency (RF) magnetron sputtering of Ge_8_Sb_92_ and Ge targets alternately. Four SLL samples, [Ge_8_Sb_92_ (15 nm)/Ge(x)]_3_ with x = 2, 5, 8 and 11 nm, and a 50 nm thick single layer film of Ge_8_Sb_92_ are prepared. All deposition processes are carried out in an Ar atmosphere at a pressure of 0.2 Pa with a flow of 30 sccm and a RF power of 20 W. An Alpha-Step 500 profiler (Tencor Instrument, Milpitas, CA, USA) was used to measure the thickness of the films.

### 2.2. Resistance Characterization of Film Crystallization

The resistance as a function of temperature (*R*~*T*) is measured in situ in a vacuum chamber where the temperature is regulated by a heater. The *R*~*T* curves are acquired at a heating rate of 10 °C/min with a TP 94 temperature controller (Linkam Scientific Instruments Ltd., Surrey, UK).

### 2.3. Coherent Phonon Dynamic Characterization of Film Crystallization

Time-resolved pump-probe differential transmission spectroscopy is used to study the coherent phonon dynamics of the samples [19,29,30]. The femtosecond laser pulses are generated from a home-made Kerr lens mode-lock Ti:sapphire oscillator and have a repetition rate of 94 MHz, a central wavelength at 840 nm and a duration of ~60 fs. The laser pulses are directed into a typical pump-probe setup and split into a pair of pulses, a stronger pulse as pump and a weaker pulse as probe with >15 intensity ratio of the pump to probe. The pump and probe pulses transmit through a convex lens of 50 mm focal length and are focused to the same area on the surface of the samples. The probe transmitted through the sample is detected by a Si photodiode whose output electrical signal is sent to a lock-in amplifier so that the transient differential transmission change of the probe can be measured. An optical chopper is used to modulate the train of pump pulses at ~1.3 kHz and synchronize the lock-in amplifier.

Each transient differential transmission profile is taken on a fresh spot that is first irradiated by a given laser irradiation fluence. It is worth noting that the laser irradiation to a fresh spot on the amorphous sample film is also performed with the pump pulses by first increasing pump laser fluence to some higher level and irradiating the fresh spot, and then reducing pump fluence down to a low level of 0.029 mJ/cm^2^. Then, in situ pump-probe measurements are made under the excitation of the low pump fluence of 0.029 mJ/cm^2^.

### 2.4. Raman Spectral Characterization of Stress in SLL Films

A Renishaw micro-Raman back-scattering spectroscopy system (Renishaw plc, Gloucestershire, UK) is used to measure Raman spectra of all samples under the excitation of a He-Ne laser (632.8 nm). The shift of Raman scattering peak positions reveals the presence and magnitude of stresses in SLL nanofilms.

## 3. Results and Discussion

**Figure 1:**
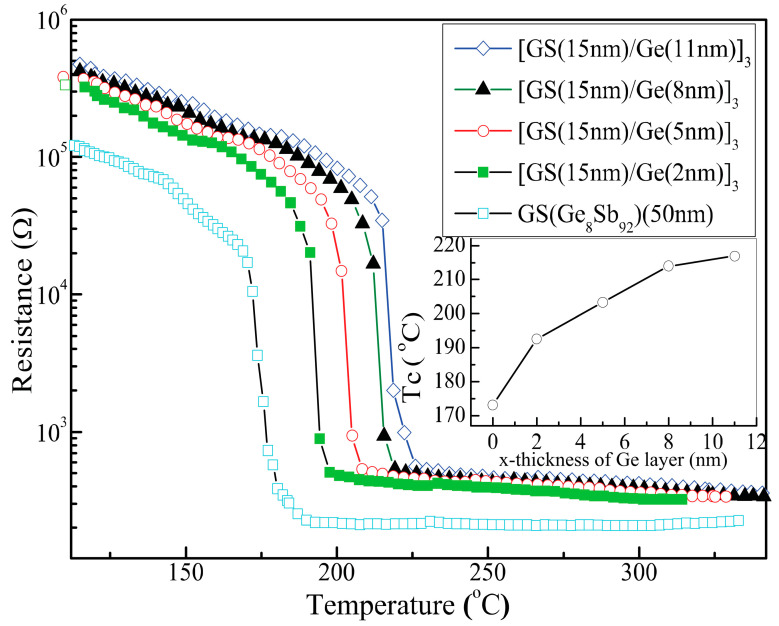
Sheet resistance of four superlattice-like (SLL) films [Ge_8_Sb_92_(15 nm)/Ge(x nm)]_3_ (x = 2, 5, 8, 11) and a single layer film Ge_8_Sb_92_ (50 nm) as a function of temperature. In the legend, GS denotes Ge_8_Sb_92_ films.

The crystallization behaviors of four SLL phase-change films, [Ge_8_Sb_92_(15 nm)/Ge (x)]_3_ with x = 2, 5, 8 and 11 nm, and one single layer Ge_8_Sb_92_ film with a thickness of 50 nm, are first investigated by measuring the sheet resistance (*R*) as a function of temperature (*T*) when the films are heated at a rate of 10 °C/min [16,17,18]. The *R~T* curves are plotted in Figure 1 for the five samples. One can see that all curves show similar change features. The sheet resistance first experiences a slow decrease with increasing temperature, and then drops sharply at some critical temperature followed by a very slow decreasing process. The sharp dropping edge shows the occurrence of crystallization. The middle point of the sharp dropping edge gives the crystallization temperature (*T_C_*) of amorphous phase-change films. All curves in Figure 1 show an initial high resistance, which implies that as-grown films are in amorphous states. It is worth noting that the crystallization of SLL [Ge_8_Sb_92_/Ge (x)]_3_ films should only take place in Ge_8_Sb_92_ layers, whereas Ge layers should mostly maintain amorphous states due to a higher crystallization temperature of amorphous Ge films [31].

One can find that the single layer 50 nm-thick Ge_8_Sb_92_ film has a crystallization temperature *T*_C_ of ~172 °C, which agrees well with an experimental report [32], where Wu et al. studied the film thickness dependence of *T*_C_ and crystallization time (*t*_C_) of a single layer Ge_8_Sb_92_ film. They found *T*_C_ and *t*_C_ increased with thinning film. As a result, it was difficult to simultaneously optimize the *T*_C_ and *t*_C_ of the single layer Ge_8_Sb_92_ film by only thinning film thickness. However, they found that 13 nm-thick Ge_8_Sb_92_ film could significantly raise *T*_C_ with respect to 50 nm-thick Ge_8_Sb_92_ film, but it did not obviously prolong *t*_C_. Consequently, one 15 nm-thick Ge_8_Sb_92_ layer is adopted in our four SLL samples to raise *T*_C_ obviously but keep *t*_C_ almost constant. On the other hand, for our four SLL samples, three periods are adopted to make the total thickness of the Ge_8_Sb_92_ layer in the four SLL samples close to 50 nm, and the total thickness of SLL film larger than 50 nm. In this way, we can test if the interfacial effects model is valid in SLL structure films. One can note from the inset of Figure 1 that four SLL samples have a lowest *T*_C_ of ~193 °C, obviously higher than the *T*_C_ (~180 °C) of 15 nm-thick single layer Ge_8_Sb_92_ film shown in Reference [32], which indicates interlayer influence or more controllability on *T*_C_ of SLL films. It is even more worth noting that the *T*_C_ of four SLL [Ge_8_Sb_92_ (15 nm)/Ge (x)]_3_ films increases as well with x (Ge layer’s thickness), as shown in the inset of Figure 1, further revealing multiple degrees of freedom to control the *T*_C_ of SLL phase-change films. However, such a rise in *T_C_* in the four SLL samples with x cannot been explained by the interfacial effect because the interfacial effects model predicted a reduction in *T_C_* with increasing thickness of phase-change films [21,22]. However, for our four SLL [Ge_8_Sb_92_ (15 nm)/Ge (x)]_3_ samples, the thickness of the phase-change layer, Ge_8_Sb_92_, is fixed at 15 nm or 45 nm (total thickness of three periods), and hence their *T*_C_ would not be changed. On the other hand, even taking account for the change in either the total thickness of the four SLL samples or the thickness of the Ge layer alone, *T_C_* would decrease with the increase in x according to the interfacial effects model.

Next, we discuss whether another effect, the reduction in the thermal conductivity of amorphous SLL films with respect to corresponding amorphous bulk phase-change films [14], can lead to the rise in *T_C_* with the increase in x. In principle, the reduction in thermal conductivity may lead to the rise in measured *T_C_* even if the real crystallization temperature (denoted by *T*_x_) of SLL films was not changed with the structure of SLL films because in our experiments the samples are heated from the bottom side (substrate) and the sheet resistance is measured at the top side (film-vacuum interface). The reduction in thermal conductivity may result in a larger temperature difference between the bottom and top sides of the samples. When the temperature at the top side reaches *T*_x_, the temperature at the bottom side must be higher than *T*_x_, while we monitor the temperature at bottom side experimentally. Consequently, *T_C_* > *T*_x_ is possible. Furthermore, the less the thermal conductivity is, the larger the difference (*T_C_* − *T*_x_) is. Therefore, it is reasonable that the *T_C_* of the four SLL films is higher than the *T_C_* of the Ge_8_Sb_92_ layer film alone. However, the question is if the *T_C_* of the four SLL samples, [Ge_8_Sb_92_(15 nm)/Ge(x nm)]_3_, should increase with x. In other words, does the thermal conductivity of the four SLL samples reduce with increasing x? We need to know the change in the thermal conductivity of the four SLL samples with x.

The effective thermal conductivity of superlattice-like films can be expressed as [33],

(1) $$K_{eff}=\frac{L_{p}}{R_{A}+R_{B}+2R_{\mathrm{int}}-R_{\mathrm{int}}/N}$$

where *R_A_* and *R_B_* are the thermal resistances of component *A* and *B* layers in the superlattice-like structure, respectively, *R*_int_ is the thermal resistance of the interface between A and B layers, *N* is the cycle number of periodicity, and *L*_p_ is the period size of the superlattice.

Based on Equation (1), we can find that the thermal conductivity of the four SLL samples increases with x (the thickness of the Ge layer) because period size, *L*_p_, increases with increasing x from 2 to 11 nm, agreeing well with the reported increase in thermal conductivity with increasing their period size in superlattice Si/Ge and Bi_2_Te_3_/Sb_2_Te_3_ films [34,35]. The fact that both the thermal conductivity and *T_C_* increase simultaneously with x implies the realness of *T*_x_ rising of four SLL samples with the increase in x. In other words, the change in Ge layer thickness (x) indeed modulates the *T*_x_ of SLL phase-change films, [Ge_8_Sb_92_ (15 nm)/Ge(x nm)]_3_.

To further verify the rise in *T*_x_ with x, it is very necessary to provide new evidence. In the following, femtosecond laser irradiation and subsequent in situ characterization of the laser-induced crystallinity by coherent phonon spectroscopy is used to detect the change in *T*_x_ with x. Laser irradiation is non-invasive and almost thermal conductivity-independent because the laser beam penetrates and heats all layers simultaneously, so that the thermal conductivity effect is not obvious and may be negligible during the heating of femtosecond laser pulses. Consequently, real *T*_x_ can be detected by this technique, which has been described in detail in the experimental section and elsewhere [29,30].

**Figure 2:**
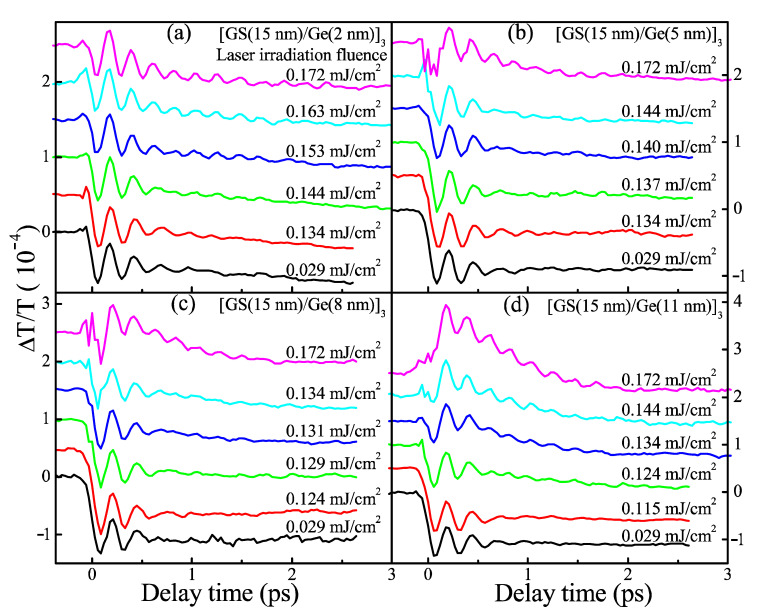
The dynamics of coherent phonon oscillation of four SLL samples measured by transient differential transmission after the irradiation of different laser fluence that is indicated over each dynamic profile. (**a**) [Ge_8_Sb_92_(15 nm)/Ge(2 nm)]_3_. (**b**) [Ge_8_Sb_92_(15 nm)/Ge(5 nm)]_3_. (**c**) [Ge_8_Sb_92_(15 nm)/Ge(8 nm)]_3_. (**d**) [Ge_8_Sb_92_(15 nm)/Ge(11 nm)]_3_. All measurements are taken under a same low pump fluence of 0.029 mJ/cm^2^. The curves are shifted vertically for clarity.

**Figure 3:**
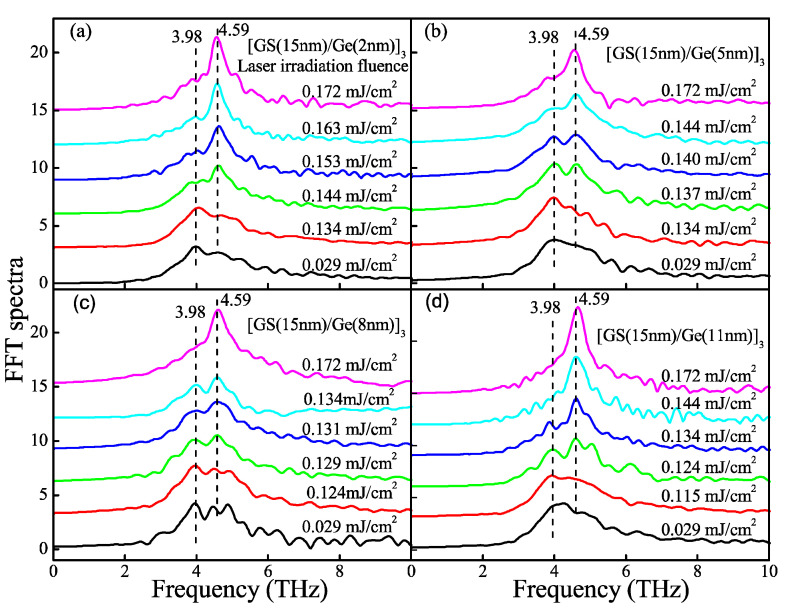
Fast Fourier transform (FFT) spectra of coherent phonon oscillations corresponding to the dynamics in Figure 2. (**a**) [Ge_8_Sb_92_(15 nm)/Ge(2 nm)]_3_. (**b**) [Ge_8_Sb_92_(15 nm)/Ge(5 nm)]_3_. (**c**) [Ge_8_Sb_92_(15 nm)/Ge(8 nm)]_3_. (**d**) [Ge_8_Sb_92_(15 nm)/Ge(11 nm)]_3_. The curves are shifted vertically for clarity.

Figure 2 shows the dynamics of coherent phonon oscillation (CPO) of four SLL films after the irradiation of different laser fluence. Their evolution should reflect the change in crystallinity induced by different laser irradiation fluence (LIF) because all dynamic profiles are measured under a same low pump fluence of 0.029 mJ/cm^2^ to prevent any phase change. One can see that two dynamic profiles at the bottom of each of the panels in Figure 2 are almost identical, which implies that a slightly higher LIF than 0.029 mJ/cm^2^ cannot lead to phase change either. As LIF increases up to some critical value, the dynamics of CPO start to change in dephasing the time and frequency of CPO. This critical (minimum) LIF value to cause the change in CPO dynamics is called a crystallization threshold (CTh) in the text below. For example, from Figure 2a, one can discern the dynamics of CPO start to change as the LIF reaches 0.144 mJ/cm^2^, and hence the CTh of the first sample [Ge_8_Sb_92_ (15 nm)/Ge(2 nm)]_3_ is 0.144 mJ/cm^2^. Similarly, one can find the CThs of another three samples [Ge_8_Sb_92_ (15 nm)/Ge(x nm)]_3_ are 0.137, 0.129, and 0.124 mJ/cm^2^ for x = 5, 8, and 11, respectively. To show the CThs more apparently, the oscillatory components of the dynamics of CPO are retrieved and fast Fouier-transformed [29,30]. Fast Fourier transform (FFT) spectra are plotted in Figure 3. One can see unambiguously that a stronger peak starts to occur at 4.59 THz when the LIF reaches CThs of 0.144, 0.137, 0.129 and 0.124 mJ/cm^2^, respectively, for x = 2, 5, 8 and 11. The peak at 4.59 THz agrees well with the vibration frequency of the A_1g_ optical phonon mode of crystalline Sb [36], implying that laser-irradiated crystallization occurs and crystallized Sb nano-crystallites occur in Ge_8_Sb_92_ layers. The above optical experimental results show that the CTh of four SLL samples decreases with the increase in x. It seems to intuitively imply that the *T*_x_ of four SLL samples decreases with increasing x. This is contradictory to the results shown in the inset of Figure 1, where *T*_x_ or *T_C_* increases with x. How can we solve this discrepancy?

First of all, we need to remind readers to notice that the CTh given by coherent phonon spectroscopy is a total laser fluence incident on samples. The total laser fluence is only partially absorbed by samples, whereas the other part of the total laser fluence is lost due to the transmission and reflection of the samples. The structures of our four SLL samples are different, and hence, they should have a different loss of transmission and reflection due to the inter- and intra-layer multiple beam interferences in SLL multilayer phase-change films. The true contribution to the laser-irradiated crystallization of samples is only from the absorbed part of the total laser fluence, but not total laser fluence incident on samples. Therefore, we must calculate the true absorbed laser fluence corresponding to CTh, called absorbed CTh (a-CTh) below. Only a-CTh is directly related to the *T_x_* of SLL films.

Based on the calculation model of reflection and transmission in multilayer films [37,38], the reflectivity and transmissivity of four SLL samples are calculated and listed in Table 1. The optical constants of the Ge_8_Sb_92_ and Ge layers used in the calculations are taken from Refs. [39,40] and listed in the last row of Table 1. One can see that the reflectivity (*R*) of SLL samples is very sensitive to the thickness change in the Ge layer and decreases significantly with the increase in x, whereas the transmissivity (*T*) of the samples is insensitive to the change in x. Consequently, the absorbance (*A = 1* − *T* − *R*) of the samples becomes sensitive to the change in x. As shown in Table 1, *A* increases so markedly with x that the really absorbed CTh (a-CTh = CTh*A) increases with x, as the data show in the seventh column of Table 1. The increase in a-CTh with x agrees well with the increase in *T*_C_ shown in the inset of Figure 1. Moreover, to know the really absorbed fluence of the Ge_8_Sb_92_ layer more accurately, we can compute the absorbance (A_1th-GS_) of the first Ge_8_Sb_92_ layer in SLL films [37]. As the data show in the sixth column of Table 1, A_1th-GS_ increases significantly with x. The increase in A_1th-GS_ with x in turn leads to the increase in the really absorbed threshold fluence of the first Ge_8_Sb_92_ layer, a-CTh_1th-GS_ = A_1th-GS_*CTh, as the data show in the last column of Table 1. The increase in a-CTh_1th-GS_ with x sufficiently proves the rising of *T_x_* with x. In other words, the crystallization temperature of four SLL samples indeed rises with the increase in Ge layer thickness. This agrees well with the increase in *T*_C_ with x revealed by the *R~T* curves in the inset in Figure 1.

Both optical and *R*~*T* measurements reveal the increase in *T*_x_ of four SLL samples with x. However, this phenomenon can be explained neither by interfacial effect nor by the reduction in thermal conductivity. It implies that some new effects exist in SLL phase-change films. Both the interfacial effect and the reduction in thermal conductivity only consider the local atomic interactions and phonon-interface scattering near the interfaces, only considering short-range interactions but ignoring possible long-range interaction, such as the stress effect. Stress is a long-range force and is ubiquitous in film materials due to lattice mismatch between two different constituent layers in SLL films and between sample films and substrates. Stress is influenced not only by film thickness [22,41,42], but also the cycle number of periodicity in SLL films [43], which is an apparent long-range effect. As a result, in our case, the variation in the Ge layer’s thickness may change the stress in SLL films, [Ge_8_Sb_92_/Ge(x)]_3_ (x = 2, 5, 8 and 11 nm). It was usual that stress reduced with the increase in the film thickness. It was already reported that stress could enhance nucleation or reduce crystallization temperature [26,27,28]. Consequently, the rise in crystallization temperature with increasing x can be explained by the reduction in stress with the increase in x. Therefore, what we need to do so far is to show the presence and the reduction in the stress with increasing x in our SLL films, [Ge_8_Sb_92_/Ge(x)]_3_.

The measurement of stresses in films is a quite challenging task. The currently reported main methods to measure stresses in thin films include beam curvature, Raman scattering spectroscopy, and X-ray diffraction [42]. Raman scattering spectroscopy is considered a simple and reliable method to measure stresses in thin films and has been widely applied in the measurement of stresses in thin films and superlattice films [41,43,44,45,46,47]. The measurement of stresses with Raman scattering spectroscopy is based on the shift of a Raman scattering peak in stressed films with respect to the Raman scattering peak in stress-free films [41].

**Figure 4:**
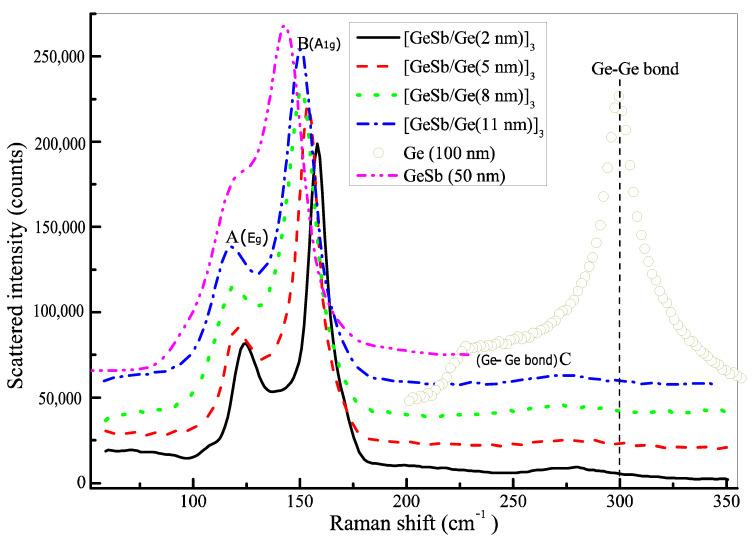
Raman spectra of four SLL films with different thickness of Ge layers and a 100 nm thick pure Ge film. The baseline of all curves is shifted upward for clarity except for the solid line curve.

We have carried out the Raman spectrum measurements of all samples. All Raman scattering spectra are plotted in Figure 4.

One can see that the Raman spectra of four SLL samples [Ge_8_Sb_92_/Ge(x)]_3_ present two stronger peaks at around 110 cm^−1^ (A) and 150 cm^−1^ (B) and a weaker broad band over a range of ~200 cm^−1^ to 350 cm^−1^ where a weak peak appears near 280 cm^−1^ (C), while the Raman spectrum of the sole thick Ge film shows a single stronger peak at 300 cm^−1^ that might be attributed to the vibration of Ge–Ge bonds [48]. The Raman spectrum of 50 nm thick Ge_8_Sb_92_ film is also plotted in Figure 4 for a contrast to the Raman spectra of four SLL films. It displays strong A and B peaks, revealing that A and B peaks in four SLL films originate from the Ge_8_Sb_92_ layers. One can also see an obvious red shift of A and B peaks with increasing thickness of the Ge layer toward A and B peaks in 50 nm thick Ge_8_Sb_92_ film.

**Figure 5:**
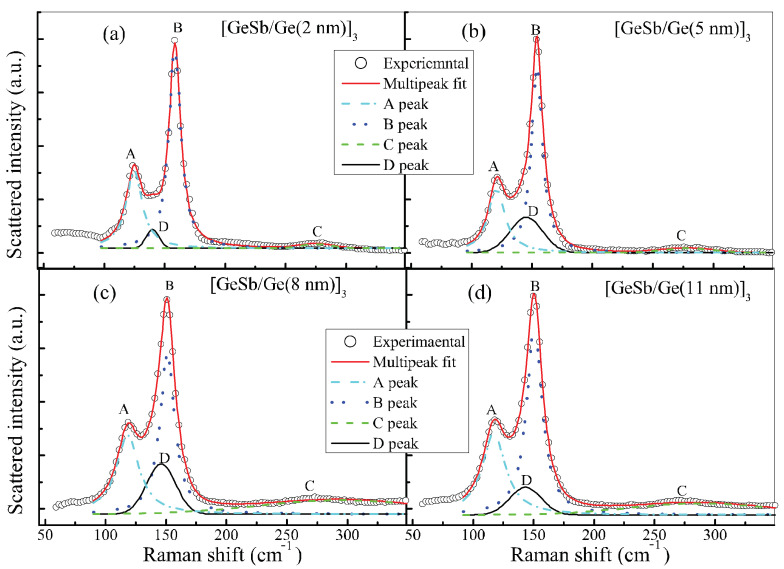
Raman spectra of four SLL sample [GS(15 nm)/Ge(x nm)]_3_ films and their best fittings with a multi-function. (**a)** x = 2, (**b**) x = 5, (**c**) x = 8, (**d**) x = 11. The letters A–D denote the individual Raman scattering spectrum of four vibration modes mentioned in the text, respectively.

To distinguish emerging vibration modes from Raman spectra, it is necessary to decompose Raman spectra by fitting Raman spectra with a multi-peak function, as usually performed in previous studies. One can see that there are three apparent peaks, A, B, and C, in Figure 4. As a result, a three peak function is first used to fit Raman spectra. However, we find peak A and the deep dip between peaks A and B cannot be fit well. As a result, we fit the spectra with a four peak function, including two Lorentz and two Gauss functions. Fortunately, Raman spectra can be fit very well by the four peak function. Raman spectra and their best fittings are plotted in Figure 5 for the four SLL sample films.

**Figure 6:**
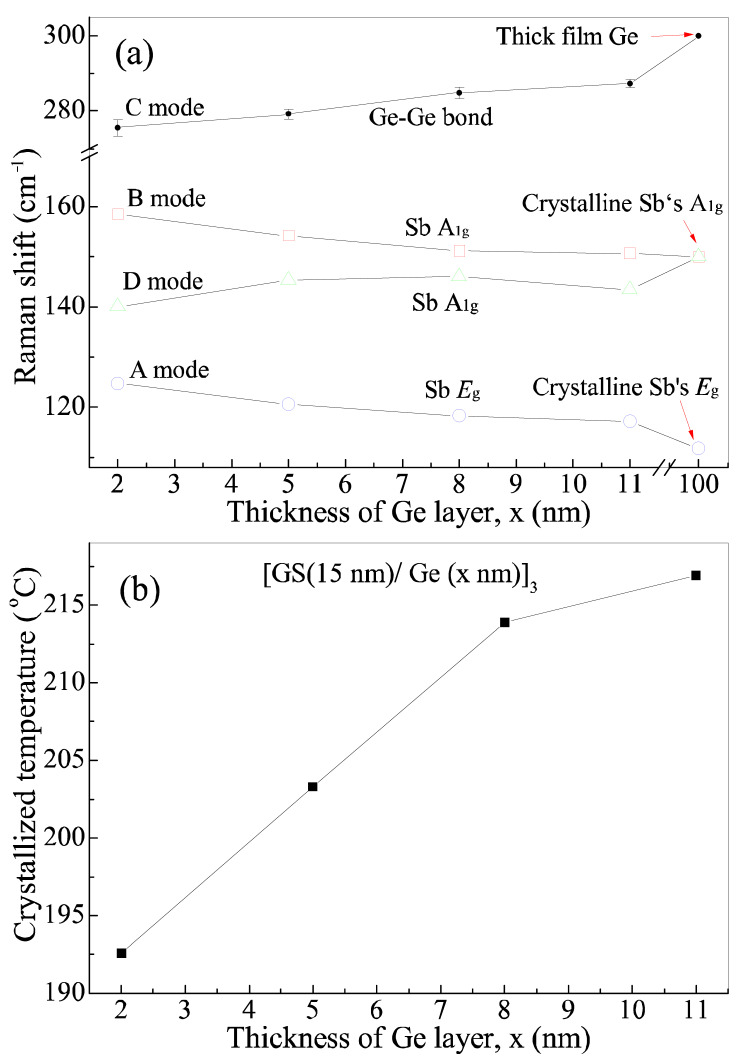
Variation in vibration frequency of various phonon modes (**a**) and crystallization temperature (**b**) with x in SLL [Ge_8_Sb_92_/Ge(x m)]_3_ films.

One can see that Raman spectra (open circles) agree very well with the four peak function (solid line). The single Raman scattering spectrum of four vibration modes decomposed by the fitting is also plotted together in Figure 5 and labeled by letters A–D, respectively. One can see that an additional D peak (black solid line) appears between peaks A and B. We find that the vibration frequency of A mode decreases from 124 to 117 cm^−1^ with increasing x, while that of B mode also decreases from 158 to 150 cm^−1^. Contrarily, the vibration frequency of C mode increases from 275 to 287 cm^−1^ with increasing x, while that of D mode also increases from 140 to 143 cm^−1^. The vibration frequencies of the four modes labeled by A–D are plotted in Figure 6a as a function of x.

One can see that the vibration frequency of A and B modes reduces monotonously with the increase in x and approaches the frequency (111.7 cm^−1^) of the *E*_g_ mode and the frequency (150 cm^−1^) of the *A*_1g_ mode I stress-free crystalline Sb, respectively [36]. Hence A and B modes are assigned to the *E*_g_ mode and *A*_1g_ of crystalline Sb in the Ge_8_Sb_92_ layer, respectively. The vibration frequency of C mode increases monotonously with increasing x and approaches the vibration frequency (300 cm^−1^) of the Ge–Ge bond in 100 nm thick Ge film, and thereby, it is attributed to the vibration mode of Ge–Ge bonds [48]. The frequency shift of phonon modes with film thickness is a typical feature of stress effect, and is studied widely in various films [35,43,44,45,46,47]. Consequently, our Raman experiments confirm the presence of stress in our SLL films. One can see from Figure 6a that the vibration frequency of mode C (Ge–Ge bond) in the Ge layer of four SLL samples is always lower than one of Ge–Ge bonds in stress-free thick Ge film or crystalline Ge, implying the presence of tensile stress in the Ge layer [48,49]. The vibration frequencies of A and B modes in Ge_8_Sb_92_ layers are always higher than the vibration frequencies, 111.6 and 150 cm^−1^, of corresponding modes, respectively, in stress-free crystalline Sb, suggesting the existence of compressive stress in Ge_8_Sb_92_ layers. Such tensile/compressive stress coupling originates from lattice mismatch between Ge_8_Sb_92_ and Ge layers in SLL films. Furthermore, the fact that the vibration frequencies of modes A, B, and C vary monotonously with x reveals the monotonous variation of stress in SLL [Ge_8_Sb_92_/Ge (x nm)]_3_ films with x. Based on the effect of the stress-enhanced crystallization reported [26,27,28], the crystallization temperature of SLL [Ge_8_Sb_92_/Ge (x nm)]_3_ films should increase monotonously with the increase in x, which agrees very well with the experimentally measured variation of crystallization temperature shown in Figure 6b and the inset in Figure 1. Therefore, the variation of crystallization temperature with x in SLL [Ge_8_Sb_92_/Ge (x nm)]_3_ films can be explained well by a new stress effect, instead of an existing interfacial effect and the reduction in thermal conductivity. Our results show that the stress in SLL films can significantly affect the crystallization temperature of SLL phase-change films. It cannot be ignored and must be considered as a new mechanism to manipulate the crystallization behaviors of nanostructure SLL phase-change films. Our new viewpoint on stress mechanism is also supported by a report [50]. Zhou et al. proposed that stress was intentionally introduced into SLL phase-change films [GeTe/Sb_2_Te_3_]n to lower melting temperature and further lower the RESET power consumption [50]. Based on our stress mechanism, the effect of the cycle number of periods on the crystallization temperature of SLL films can also be explained well because it was reported that the variation of cycle number in SLL films could lead to the accumulation and release of stresses [19,43]. Up to date, the stress and interfacial effects should coexist in SLL phase-change films. The two effects usually become stronger with thinning films, but they affect crystallization temperature oppositely. In other words, the interfacial effect increases the crystallization temperature with decreasing x, whereas the stress mechanism lowers it. Consequently, actual variation of the crystallization temperature of SLL films will depend on the competition between the two effects. Actual crystallization temperature will rise with decreasing x if the interfacial effect prevails over the stress effect. Contrarily, the crystallization temperature will become lower with decreasing phase-change film thickness if the stress effect is dominant. Maybe it was just such a case reported in Ref. [8], where the crystallization temperature of two sandwiched structures, Al/GeSb (x nm)/Al and W/GeSb (x nm) /W, rises with increasing x. Therefore, previously reported anomalous change in crystallization temperature with the film thickness and cycle number of periods in SLL films can be explained well qualitatively based on the stress effect prevailing over the interfacial effect [8,19].

Finally, we discuss the possible origin of mode D in Figure 6a. One can see that the vibration frequency of D mode is always lower than but approaches one of crystalline Sb’s *A*_1g_ mode with increasing x. As a result, we speculate that D mode still results from the *A*_1g_ mode of Sb crystallites in Ge_8_Sb_92_ layers, but it is influenced by tensile stress, unlike B mode which is influenced by compressive stress. This case is possible because the stress in the Ge_8_Sb_92_ layers may be biaxial. The stress along one axis is compressive, while it is tensile along the other axis. Sb crystallites in Ge_8_Sb_92_ layers are formed from excess Sb exceeding stoichiometric content in Ge_8_Sb_92_ layers, and they orientate randomly. Consequently, partial *A*_1g_ modes suffer from the compressive effect, and they behave like A mode, but the other part of *A*_1g_ modes experiences tensile stress and behaves like D mode.

## 4. Conclusions

We have studied the crystallization behaviors of four SLL phase-change films, [Ge_8_Sb_92_ (15 nm)/Ge(x nm)]_3_ with x = 2, 5, 8, and 11, by using both conventional static heating and transient femtosecond laser irradiation, respectively, followed by measurements of sheet resistance and coherent phonon spectroscopy. Two kinds of measurements reveal the anomalous increase in the crystallization temperature of SLL phase-change films with x. However, this increase cannot be explained by existing opinions, interface effects and the reduction in thermal conductivity, implying the presence of new effects in SLL films. Interfacial effects considered only local atomic interactions near the interface between two component layers, while the reduction in thermal conductivity performed the phonon-interface scattering effect. Both of them ignore a very important and ubiquitous lattice mismatch effect in SLL films. The lattice mismatches certainly result in the occurrence of stress in SLL films—a long-range effect. It was well known that stresses could enhance the crystallization of amorphous materials. Therefore, a new stress effect is proposed to explain the anomalous increase in crystallization temperature of our four SLL films with x. Raman scattering spectroscopy is used to characterize the presence of stresses in crystallized SLL films. The Ge-Ge phonon mode in Ge layers and the *E*_g_ and *A*_1g_ phonon modes of Sb crystallites in Ge_8_Sb_92_ are observed in Raman spectra. However, Raman shifts of those phonon modes deviate obviously from the standard shift positions of respective corresponding phonon modes in stress-free crystalline films, indeed confirming the presence of stresses in crystallized SLL films. Raman shifts of the *E*_g_ and *A*_1g_ phonon modes of Sb crystallites in Ge_8_Sb_92_ layers are always larger than their respective standard shifts in stress-free Sb crystals, but they approach the standard shift values with increasing x, implying the presence of compressive stress and reduction with the increase in x. Inversely, the Raman shift of Ge-Ge mode is always less than its standard shift in stress-free Ge crystals, but it approaches the standard shift values with increasing x, suggesting the presence of tensile stress and reduction with the increase in x. This compressive/tensile stress coupling between Ge_8_Sb_92_ and Ge layers reveals lattice mismatches between two adjacent layers in SLL films. Based on the stress-enhanced crystallization phenomena reported, the increase in crystallization temperature of our four SLL films with x can be explained well by stress release in SLL films with increasing x. Our results show that stress and interfacial effects coexist and compete because they change crystallization temperature oppositely. Previously reported anomalous variations in crystallization temperature with the film thickness and cycle number of periods in SLL films can be explained well by the stress mechanism. Our results provide a new degree of freedom to manipulate the crystallization behaviors of SLL phase-change films.

## Figures and Tables

**Table 1:** The calculations of the total reflectivity, transmissivity and absorbance as well as the absorbance of the first Ge_8_Sb_92_ layer of four SLL samples. Then, absorbed laser fluence thresholds of the whole sample and first Ge_8_Sb_92_ layer are calculated.

Samples	CTh Measured by Coherent Phonon Spectra (mJ/cm^2^)	Transmissivity T (%)	Reflectivity R (%)	Absorbance A = 1 – T – R (%)	Absorbance of First GS Layer, A_1th-GS_ (%)	a-CTh = CTh*A (*10^−2^ mJ/cm^2^)	a-CTh_1th-GS_ = CTh*A_1th-GS_ (*10^−2^ mJ/cm^2^)
[GS/Ge(2nm)]_3_	0.144	10.82	49.01	40.17	16.46	5.78	2.37
[GS/Ge(5nm)_3_	0.137	11.00	45.52,	43.48	18.18	5.96	2.49
[GS/Ge(8nm)]_3_	0.129	11.19	41.47	47.34	20.16	6.11	2.60
[GS/Ge(11nm)]_3_	0.124	11.36	37.09	51.55	22.28	6.39	2.76
GS denotes Ge_8_Sb_92_ (15 nm thick)		*N* = 3.50 + 2.29i, for amorphous Ge_6_Sb_94_ [39]			*N* = 3.49 + 0.25i, for amorphous Ge [40]		

## Data Availability

Please refer to suggested Data Availability Statements in section “MDPI Research Data Policies” at https://www.mdpi.com/ethics.
